# Transcranial Direct Current Stimulation as an Add-on Treatment to Cognitive-Behavior Therapy in First Episode Drug-Naïve Major Depression Patients: The ESAP Study Protocol

**DOI:** 10.3389/fpsyt.2020.563058

**Published:** 2020-11-03

**Authors:** Sandra Carvalho, Óscar F. Gonçalves, André R. Brunoni, Ana Fernandes-Gonçalves, Felipe Fregni, Jorge Leite

**Affiliations:** ^1^Psychological Neuroscience Laboratory, CIPsi, School of Psychology, University of Minho, Braga, Portugal; ^2^Department of Physical Medicine and Rehabilitation, Spaulding Neuromodulation Center, Spaulding Rehabilitation Hospital, Massachusetts General Hospital and Harvard Medical School, Boston, MA, United States; ^3^Proaction Laboratory, Faculty of Psychology and Educational Sciences, University of Coimbra, Coimbra, Portugal; ^4^Department and Institute of Psychiatry, Service of Interdisciplinary Neuromodulation, Laboratory of Neurosciences (LIM-27), Interdisciplinary Center for Applied Neuromodulation University Hospital, University of São Paulo, São Paulo, Brazil; ^5^Department of Psychiatry, CUF Porto Hospital, Porto, Portugal; ^6^Univ Portucalense, Portucalense Institute for Human Development–INPP, Porto, Portugal

**Keywords:** MDD (Major Depressive Disorder), study protocol, drug-naïve, tDCS (transcranial direct current stimulation), CBT (cognitive-behavioral therapy)

## Abstract

**Background:** Major Depressive Disorder (MDD) affects more than 264 million people worldwide. Current treatments include the use of psychotherapy and/or drugs, however ~30% of patients either do not respond to these treatments, or do not tolerate the side effects associated to the use of pharmacological interventions. Thus, it is important to study non-pharmacological interventions targeting mechanisms not directly involved with the regulation of neurotransmitters. Several studies demonstrated that transcranial Direct Current Stimulation (tDCS) can be effective for symptoms relief in MDD. However, tDCS seems to have a better effect when used as an add-on treatment to other interventions.

**Methods/Design:** This is a study protocol for a parallel, randomized, triple-blind, sham-controlled clinical trial in which a total of 90 drug-naïve, first-episode MDD patients (45 per arm) will be randomized to one of two groups to receive a 6-weeks of CBT combined with either active or sham tDCS to the dorsolateral prefrontal cortex (DLPFC). The primary outcome will depressive symptoms improvement as assessed by the Montgomery-Asberg Depression Rating Scale (MADRS) at 6-weeks. The secondary aim is to test whether CBT combined with tDCS can engage the proposed mechanistic target of restoring the prefrontal imbalance and connectivity through the bilateral modulation of the DLPFC, as assessed by changes over resting-state and emotional task eliciting EEG.

**Discussion:** This study evaluates the synergetic clinical effects of CBT and tDCS in the first episode, drug-naïve, patients with MDD. First episode MDD patients provide an interesting opportunity, as their brains were not changed by the pharmacological treatments, by the time course, or by the recurrence of MDD episodes (and other comorbidities).

**Trial Registration:** This study is registered with the United States National Library of Medicine Clinical Trials Registry (NCT03548545). Registered June 7, 2018, clinicaltrials.gov/ct2/show/NCT03548545. Protocol Version 1.

## Background

Major Depressive Disorder(MDD) is widely recognized as a staggering global healthcare challenge, as well as a potentially lethal illness (1). The worldwide prevalence of Depression is about 3.4% [2–6%], and mild forms of depression are the most prevalent−13%, as compared to 4% for moderate forms and 5.1 % for severe forms of depression (2). The prevalence in females is about 4.1% and about 2.7% in males (2). Overall, MDD is thought to affect 264 million people worldwide, thus ranking second in the most common causes of disability with prospects of becoming the first by 2040 (3).

The current standard care for MDD involves the use of psychotherapy, antidepressant medication, or a combination of both. Despite the costs involved in these interventions, the efficacy of such treatments may have been overestimated, with recent data suggesting that remission rates can be as low as 23% depending on the self-report scale used (4). Furthermore, 30% of patients suffering from MDD still exhibit depressive symptoms despite the appropriate psychological and pharmacological treatments (5). In order to overcome this, several treatments are frequently combined, usually by the use of drug augmentation and/or combination of different drugs, which often increases the risk of adverse effects (6). Thus, the development of effective treatment alternatives for MDD, which includes non-pharmacological interventions targeting mechanisms not directly involved with the regulation of neurotransmitters, is an urgent research priority. However, in order to do so, it is important to understand the underlying neural mechanisms involved in MDD.

Evidence coming from several electroencephalography (EEG) (7–9), neuroimaging (10, 11), and neuromodulation (12–15) studies showed that the dorsolateral prefrontal cortex (DLPFC) is as an important area of dysfunction in depression, mainly due its left hypo and right hyper-functioning. This inter-hemispheric imbalance over the DLPFC has been shown to be an indicator of lifetime MDD, or in conjunction with depressive self-schema (i.e., an interconnected negative internal representation of the self that has been associated to the onset and maintenance of depressive state (16, 17) to be a predictor of a first prospective MDD episode (18). Nevertheless, some studies failed to show the link between decreased left frontal activation and depression (19).

Transcranial direct current stimulation (tDCS) is a non-invasive method of brain stimulation that is capable of depolarizing or hyperpolarizing the neural membrane and as such, it has been used in people suffering from depression, by placing the anode (excitatory) over the left and the cathode (inhibitory) over the right DLPFC, or by placing the anode over the left DLPFC and the cathode over the right supra-orbital region.

For instance, in one study, 64 MDD patients were randomized to 15 sessions of 2 mA tDCS over 3 weeks, and tDCS was shown to be able to decreased MDD symptoms (20). However, in another study, not only active tDCS was superior to sham but also the combination of tDCS with sertraline was significantly more effective in reducing depressive symptoms than either treatment alone (21). Although tDCS *per se* showed promising results in treating MDD, the previous trial highlights that the effects of tDCS can be enhanced by combining it with other interventions. Overall, tDCS seems to decrease MDD symptoms by a pooled effect size of 0.36 (22). Moreover, according to a recent individual patient data meta-analysis, tDCS seems to be less effective in high-resistant patients, suggesting that tDCS may be a promising add-on therapy to therapies such as the cognitive behavioral therapy (CBT) (23) or cognitive control therapy (24, 25).

CBT is an empirically validated therapy for the treatment of MDD. Several studies have demonstrated the efficacy of CTB alone (26, 27) or as adjuvant to medication (28–30) in acute depression. Another advantage of CBT over anti-depressant drugs is its long-term effects, namely protecting against relapses and recurrences after active treatments has ended (27, 31, 32). Additionally, CBT is a well-established therapy that can restore or normalize abnormal brain activity, namely prefrontal alpha activity (33). Namely, an increase in left frontal brain activity after CBT in individuals with anxiety and depression.

It is important to highlight that MDD seems to induce profound changes in the brain, namely structural alterations in fronto-cingulate-striatal circuits (34–36). However, and somewhat surprisingly, first episode MDD patients have not been extensively studied with tDCS (37, 38). These first episode MDD patients provide an interesting opportunity, as their brains were not changed by the pharmacological treatments, by the time course, or by the recurrence of MDD episodes (and other comorbidities).

## Purpose, Primary and Secondary Outcomes

Therefore, we propose to study the clinical and mechanistic effects of the combination of two well-studied interventions—CBT and tDCS—for the treatment of MDD in drug naïve first episode patients. The primary outcome will be the clinical effects (severity of depression/mood amelioration), as measured by the Montgomery-Asberg Depression Rating Scale (MADRS). Secondary outcomes will be resting state and emotional task eliciting EEG, which will be useful simultaneously to understand the neural effects of the intervention, as well as potential response predictors for future trials.

## Research Question, Aims, Hypothesis

By combining two interventions that showed promising results in MDD (CBT and tDCS), we aim to investigate the clinical and underlying neurophysiological effects in first-episode drug naïve MDD patients. The main research question underlying this proposal is whether tDCS as add-on therapy to CBT in drug naïve, first-episode MDD patients could produce greater significant clinical improvements, as measured by the MADRS, when compared to CBT alone. We hypothesize that the combination of these therapies will produce a synergetic effect in the brain, potentiating the effects of CBT and decreasing the depressive symptomatology as assessed by the MADRS.

A secondary aim will be to perform the dose calculation (number of sessions) required to induce a clinically significant effect (50% decrease in the MADRS score). The underlying hypothesis is that the combination of tDCS with CBT will require a lesser number of sessions in order to elicit this clinical meaningful effect.

The second major scientific question is whether these clinical improvements will be correlated with the rebalancing of the inter-hemispheric asymmetry of EEG alpha activity toward the left hemisphere, as assessed by resting-state and emotional task eliciting-EEG. Here, the hypothesis is that both interventions (combined and alone) will reduce the inter-hemispheric alpha imbalance, as indexed by EEG power; however, that reduction will be more pronounced in the group that received the add-on intervention, as compared to CBT alone.

## Methods

### Trial Design, Setting and Registration

**Figure 1:**
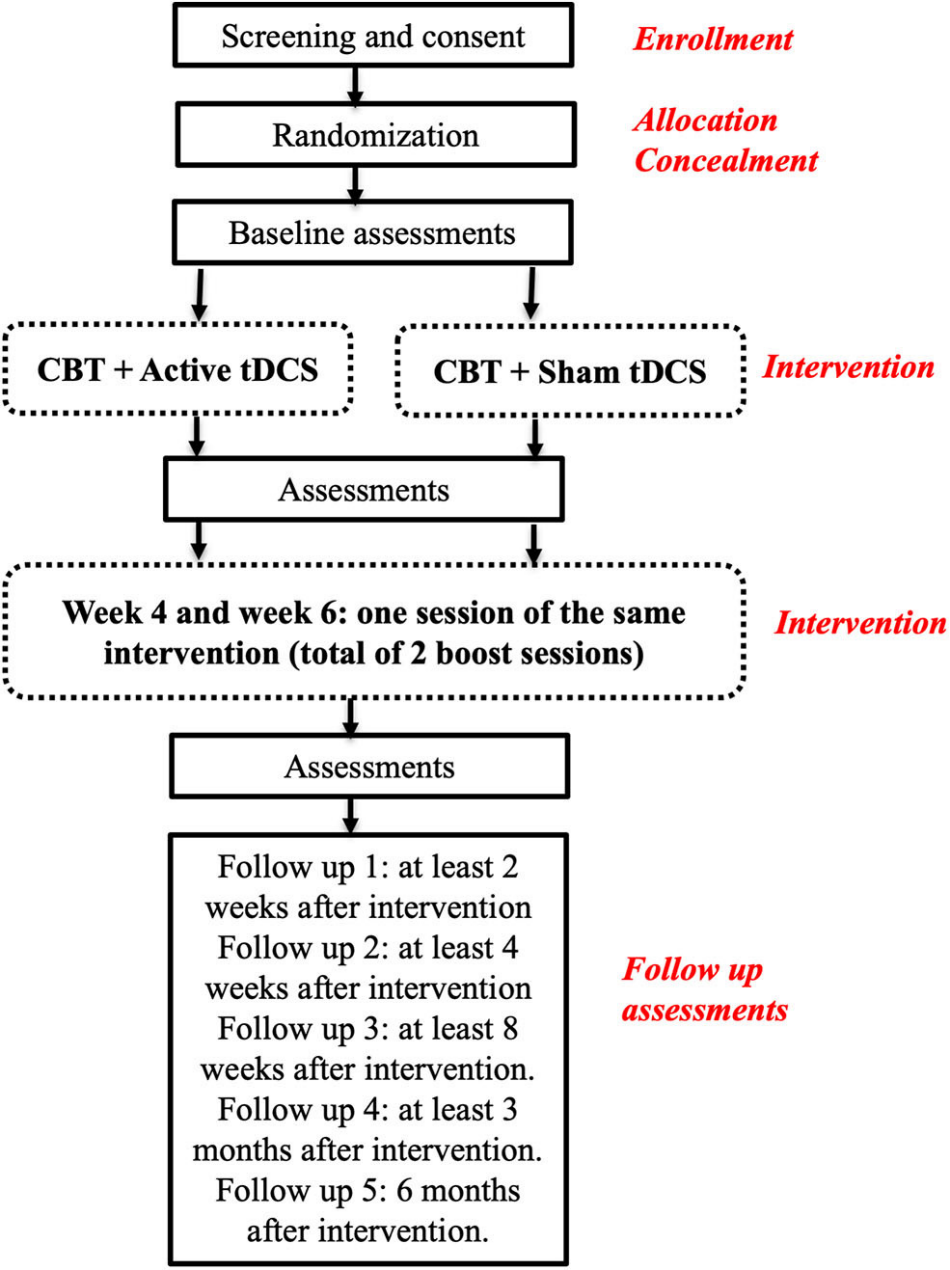
Overview of the Study design (tDCS, transcranial Direct Current Stimulation; CBT, Cognitive Behavioral Therapy).

This is a parallel, randomized, triple-blind, sham controlled clinical protocol in which a total of 90 drug-naïve, first-episode MDD outpatients (45 per arm) will be randomized to one of two groups: active bilateral tDCS over the DLPFC combined with CBT or sham tDCS combined with CBT (Figure 1).

**Figure 2:**
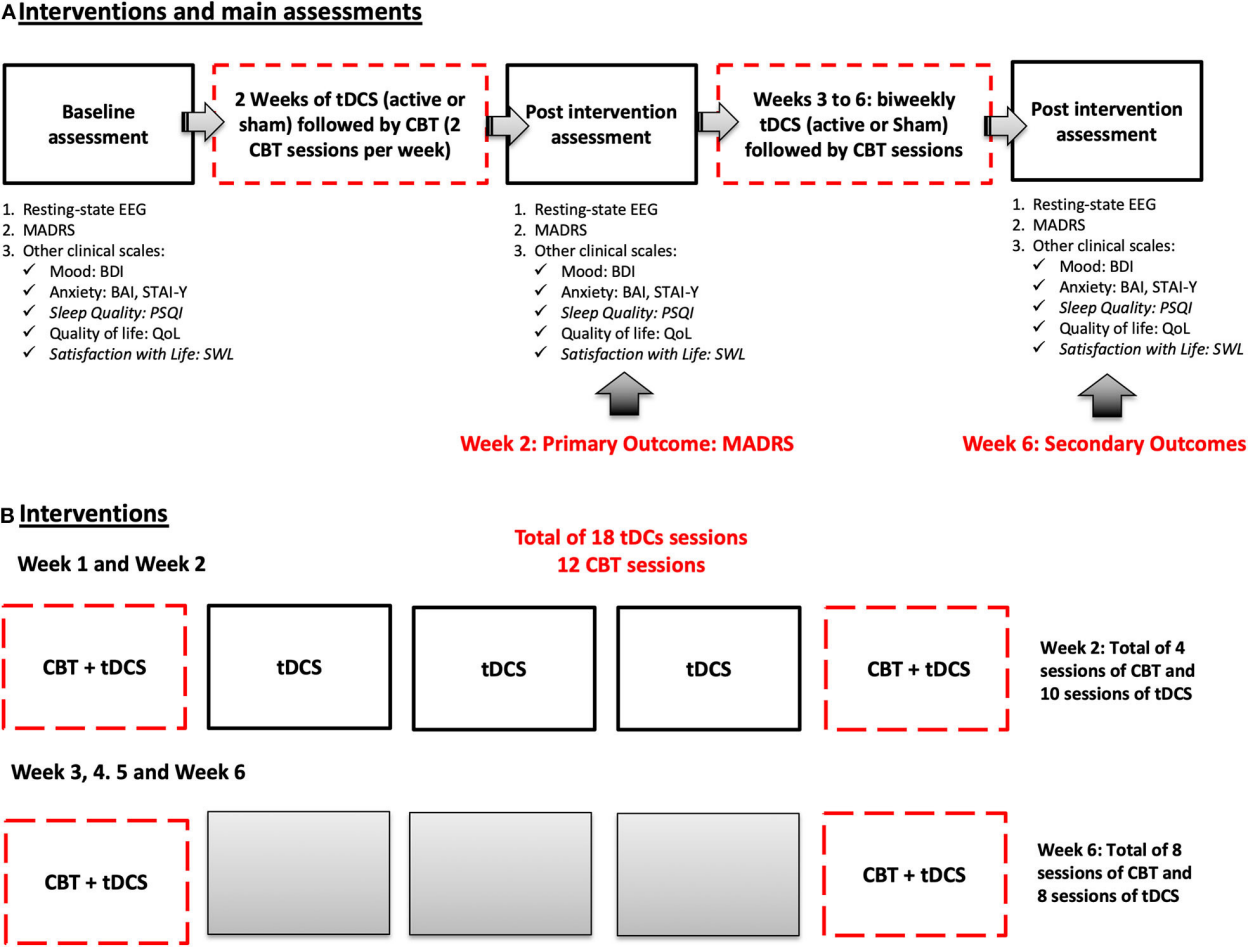
Schematic representation of the main phases of the trial. **(A)** Shows the timeline of assessments. **(B)** Details how interventions sessions will be administered by week.

Patients will complete a 6-weeks treatment that involves 18 tDCS sessions (active or sham) and 12 CBT sessions. For the first 2 weeks of intervention, they will receive 10 daily sessions of tDCS (from Monday to Friday) and 4 sessions of CBT (combined at same time with tDCS for the first 30 min, on Monday and on Friday). They will then receive two booster sessions of tDCS combined with CBT will be on weeks 3, 4, 5, and 6 (Figure 2B).

This study will then be organized in 3 distinct phases. Phase 1: Recruitment and baseline assessments; Phase 2: Intervention; Phase 3: Post-treatment assessment and 6-month follow-up (Figure 1). Once eligibility is determined and consent provided, patients will be randomly assigned to one of the two groups in a 1:1 allocation ratio, by the means of a list generated by an automatic web-based randomization program.

After an initial baseline assessment (visit 1) (for more details see task 1), patients will receive 10 consecutive sessions (in two weeks) of active or sham tDCS (2 mA, 30 min), and 6 sessions of CBT every other day (3 CBT sessions per week). Patients may fail 1 or 2 sessions of tDCS and/or CBT; and in that case, they will have the opportunity to receive the missing session (s) in the following week. The primary outcome will be assessed by week 2, using MADRS. Other assessments will be also performed by week 2, such as EEG, and other clinical measures (see Table 1). By week 4 and 6, patients will receive additional booster sessions of the same intervention (active or sham tDCS followed by CBT). Patients will perform the same assessments on week 6 (Figures 2A,B). tDCS sessions will follow the same design previously tested by two members of our group, that showed significant clinical effects of tDCS alone, and tDCS combined with sertraline in moderate to severe MDD (21).

**Table 1:** Summary and timeline of assessments.

	**Consent and Screening**	**Assessment pre-intervention**	**EEG pre-intervention**	**Interventions Session and tDCS**	**Assessment 15 days**	**EEG 15 days**	**Interventions Session and tDCS**	**Assessment post-intervention**	**EEG post-intervention**	**Follow-ups**	
				***Daily Sessions***			***Bi-weekly sessions***			***15 day, 1, 2, 3, and 5 months***	
	**Email / Visit 1**	**Visit 1**	**Visit 2**	**Visit 3-12**	**Visit 13**		**Visit 14-21**	**Visit 22**		**Visit 24 – 27**	
***Approximate visit time***	***1 h 30 min***	***30 min***	***1 h***	***50 min***	***30 min***		***50 min***	***30 min***		***30 min***	
**PRESCREENING FOR ELIGIBILITY, CONSENTING, AND CLINICAL INTERVIEW**											
Pre-screening questionnaires (inclusion and exclusion criteria)	X										
Consent Informed (2 copies)	X										
Questionnaire eligibility for the study	X										
Questionnaire eligibility to tDCS	X										
Questionnaire eligibility to EEG	X										
Demographics and medical questionnaire		X									
The structured clinical interview for DSM-5 (SCID-V)		X									
**PRIMARY AND SECONDARY OUTCOME ASSESSMENTS**											
The satisfaction with life scale (SWL)		X			X			X		X	
The pittsburgh sleep quality index (PSQI)		X			X			X		X	
State-trait anxiety inventory (STAI-Y)		X			X			X		X	
Beck anxiety inventory (BAI)		X			X			X		X	
Beck's depression inventory (BDI)		X			X			X		X	
Montgomery-asberg depression rating scale (MADRS)		X			X			X		X	
Blinding questionnaire								X			
Visual analog scales (VAS)—pre and post				X			X				

Structured CBT sessions will follow the NICE guidelines from the National Institute for Health and Care Excellence (39). Patients will receive a total of 12 CBT sessions administered biweekly for 6 weeks. Mood, will be evaluated at the end of each week, as to perform the dose calculation (i.e., number of sessions) required to induce a clinical significant effect of at least 50% decrease in the MADRS scores.

In addition to the primary outcome measure (MADRS), secondary outcome measures will be clinical response as measured by scores on the 17-item Hamilton Depression Rating Scale, Beck Depression Inventory (BDI), Beck Anxiety Inventory (BAI), clinician-rated Clinical Global Impression–Severity of Illness scale, and quality of life (QoL). These scales will be used at baseline, week 2, 4, 6, and follow-ups (up to 6-month follow up).

Resting-state EGG assessments will be performed on baseline session, on weeks 2 and 6. These assessments will allow us to assess the neural effects of these interventions in the brain.

The Standard Protocol Items: recommendations for Interventional Trials (SPIRIT) statement were used as a framework for developing the study methodology for this trial (40, 41).

### Participants

A total of 90 drug-naïve, first-episode MDD outpatients (45 per arm) will be randomized to receive either CBT combined with active bilateral tDCS over the DLPFC or CBT combined with sham tDCS.

### Eligibility Criteria

Participants will be included in this study if they meet the following criteria: 1) Aged 18–75 years; 2) Unipolar, nonpsychotic MDD (DSM-V); 3) Score in the MADRS 7 and above (mild, moderate, and severe depression); 4) Low risk of suicide, as evaluated during the clinical interview and through the Scale for Suicidal Ideation [Mild to Moderate SIS; (42)]; and 5) Able to sign informed consent.

Potential participants meeting any of the following criteria will be excluded: 1) any contraindication to receive tDCS (such as metal in the head, implanted brain medical devices); 2) any significant or unstable neurologic or psychiatric disorder other than MDD, such as epilepsy, Parkinson's Disease, Dementias, eating disorders, OCD Spectrum disorders, among others); 3) history of substance abuse within the past 6-months, 4) Any personality disorders; or 5) any severe life-threatening disorders or concurrent medical condition likely to worsen patient's functional status in next 6-months such as; cancer, or severe heart, kidney, or liver diseases. Participants with reported high risk of suicide will be excluded from the study and will be recommended to receive support from an experience and licensed psychologist/psychiatrist. Information about local and national institutions that provide support to cope with suicidal behaviors and though will be provided.

A screening questionnaire that addresses the specific inclusion and exclusion criteria will be applied to each participant prior to the SCID-5 interview and will help to screen out participants. Diagnosis will be performed using the Structured Clinical Interview for DSM-5 (SCID-5) (43, 44), a widely used semi structured clinical interview designed to evaluate psychopathology, following the categories in the DSM 5 (Diagnostic and Statistical Manual of Mental Disorders, Fifth Edition) for the clinical use and clinical research. Potential participants will also answer the tDCS eligibility questionnaire and the SIS to evaluate the likelihood of suicide attempt. Patients with high likelihood of suicide attempt will be not included in our study and will be recommend for psychological and/or psychiatric intervention.

### Description of the Interventions

#### Cognitive-Behavioral Therapy (CBT)

CBT will be performed following the structure proposed by Beck et al. (17, 45). Each session will last 60 min and will be designed individually for each patient, according to the severity of Depression—including behavioral activation and problem-solving techniques. A licensed and trained practitioner will be responsible for facilitating the self-help programme and for reviewing progresses and outcomes during psychotherapy sessions. A senior clinical psychologist with extensive experience in CBT in patients with depression will supervise the clinical work. A clinical meeting will be hosted weekly in order to perform quality assurance of the therapeutic process.

#### Transcranial Direct Current Stimulation (tDCS)

tDCS will be delivered by an Eldith DC Stimulator Plus (Neuroconn, Germany), using 25 cm^2^ saline-soaked electrode sponges. Anode will be placed over the left (F3) and cathode over the right DLPFC (F4). For the active tDCS, participants will receive 2 mA (current density = 0.80 A/m^2^; with 15/15 s ramp/ramp down) for 30 min/d. For sham tDCS, patients will receive 15 s of 2 mA intensity, and 15/15 s ramp in and ramp down, with the same montage of electrodes; however, the device will be turned off after 45 s of active stimulation. Each tDCS session will last about 40 min: 30 min of stimulation and 10-min of set up. tDCS sessions will be performed by a research assistant, not involved in the CBT sessions.

### Description of the Assessments

These instruments will be used at baseline, after the first 2 weeks of intervention, at the end of the 5-weeks of biweekly sessions and follow-up visits (up to 2 months after the intervention period) as detailed in the table below. Please see Table 1 for the complete assessments timetable.

### Outcomes

#### Primary Outcome

For the depression assessment, we will use the Montgomery-Asberg Depression Scale (MADRS) as primary outcomes. Criteria for the use of this clinical scale will be the same as we previously used in Brunoni et al. (21). Secondary outcomes will include also the clinical response (categorical variable, defined as > 50% reduction of the baseline MADRS score), clinical remission (categorical variable, defined as a MADRS scores ≤ 10), and scores on the BDI.

#### Secondary Outcomes

Resting state EEG and task-elicited prefrontal EEG alpha asymmetry—Resting state EEG screening will be carried out before the intervention (baseline), and in the end of each week of intervention. Each screening will comprise a resting state EEG (3 min eyes open and 3 min eyes closed) and a task-related EEG data collection. The task-related screening will last about 3-min in an open-eyes active state. For this task, we will use a facial emotion task, with approach and avoidance facial expressions, similar to the task used by Stewart and colleagues

EEG will be acquired using 20 channel Starstim (Neuroelectrics, Barcelona, Spain), following the 10/20 system, in a continuous mode at a digitization rate of 500 Hz, with a bandpass filter of 0.01–100 Hz. Electrode impedances will be kept below 5 kΩ and EOG will be recorded from two additional bipolar channels. EEG data will be segmented into 1.5–3 s epochs centered on subject's responses (at least 50 epochs) using EEGlab.

### Sample Size Calculation

For the sample size calculation, we assume an effect size(d) of 0.66 [upper limit for the 95% CI of the pooled effect size of tDCS on MDD (22) that for a two-side α of 0.05 and a power of 80%, requires a total of 76 patients (38 per group). We increased the sample size by 15% to 45 per group to account for unexpected factors. This sample size will be adequate to detect this magnitude of effect.

### Recruitment

The enrollment of patients will be performed mainly from our Clinical Service at the School of Psychology, and by referral from Primary Care Physicians. After initial referral, potential patients with first episode MDD as primary diagnosis, will undergo the general inclusion and exclusion criteria check list in order to assess their potential eligibility.

We will also use social media (such as the Lab Facebook page), as well as flyers posted in specific spaces such as clinical settings (hospital, clinics), Universities, etc. The first screening will be performed by a research assistant (with a clinical Psychology degree) and the full assessment of the patient will be done by a clinician specialized in MDD (blinded to the study arm).

### Randomization

Once eligibility and consent have been approved and obtained, randomization will occur using the randomized list generated by an automatic web-based randomization program. Patients will be randomly assigned to one of the two groups in a 1:1 allocation ratio. Randomization order will be kept in sealed envelopes; therefore, patients will get their assignment according to the order of entrance in the study. This will also ensure that all patients and all other investigators are kept blind to this assignment for the duration of the study (allocation concealment) (see Figure 1, for study overview).

The randomization procedures described above will be followed for assignment to treatment groups. Following initial screening, during participant enrollment, a research associate will assign them to their randomly generated treatment group, keeping all patients and all other investigators blind to this assignment for the duration of the study.

### Blinding Procedure and Assessment

Participants, the psychologists performing the CBT, the ones performing the assessments, as well the statistician will remain blinded to the tDCS condition up to the end of the clinical trial, ensuring a triple blind design. Researchers applying tDCS will not be blinded. If a serious adverse event occurs, the Principal Investigator (PI) will be responsible for removing the blinding and notify the Ethics Committee within 24 h.

Blinding assessment will be performed to both participants and researchers who assessed the outcomes.

### Assessments

#### Eligibility and tDCS Assessments

*Questionnaire to assess eligibility to participate in the study*: this questionnaire aims to evaluate inclusion and exclusion criteria to participate in the study. It includes specific questions about neurologic and psychiatric history, history of head injuries, drugs use, and/or abuse, history of treatments, etc.

*Side Effects Questionnaire*: At each stimulation session, patients will complete a questionnaire to evaluate potential adverse effects of tDCS (tingling, burning sensation, headache, neck pain, mood alterations). If any side effects are reported, the degree of relatedness to the intervention will be assessed on a 5-point scale. This type of adverse events questionnaire has been used frequently in our previous tDCS studies (46–52) including in patients with MDD. In order to further control for changes in suicidal thoughts, we will add a specific question for suicide that can be follow-up with the SSI, if scores are equal to or >3.

*The Structured Clinical Interview for DSM 5 (SCID 5):* this is a semi-structured clinical interview (the clinician version) that guides the clinician step-by-step through the DSM-5 diagnosis process. This interview will be essential to confirm the diagnosis of first episode MDD and to evaluate for possible comorbid psychiatric disorders (43).

*Scale for suicide ideation (SSI):* this is a 19-item scale that aims to quantify and assess suicidal intention (42). Patients scoring SSI≥6 will not be enrolled in the study (53).

*tDCS blinding questionnaire*: After the treatment has ended, patients will complete a questionnaire to determine if our blinding methods were effective. We are using a 30 s sham montage, just as we use in our other trials, keeping the device on the subject for the duration of the session. The tDCS blinding questionnaire is organized in two main questions: 1. Please answer the questions to the best of your ability: 1.1 Did you receive: Sham Stimulation (tDCS) or Active Stimulation (tDCS); 1.2 Please, rate how confident you feel in your answer (please check one), from 1 (not confident at all), 2, 3 (somewhat confident), 4, to 5 (completely confident).

#### Demographic and Clinical Assessments

*Demographics information:* We will record information about the demographic characteristics of the study population such as age, gender, race, level of education, and social status.

*Medication Use Log:* Medication use information will be obtained at enrollment and updated on a weekly basis, by means of a Medication Log. Participants will record their current medications and dosages weekly, until completion of the study. Medication diaries are commonly used to record changes in medication use during the study period. We will also use the Antidepressant History Treatment Form (ATHF) to assess treatment refractoriness.

*Montgomery-Asberg Depression Scale* (MADRS): For the depression improvement assessments, we will use the MADRS for the primary outcome. This is widely used scale for the measurement of severity of depressive symptoms in patients with MDD. The scale is divided into 10 items, each scored on a 0 to 6-point ordinal scale (54). The MADRS will be administered according to a structured interview procedure that has been empirically found to result in high inter-rater reliability scores.

*Beck Depression Inventory (BDI):* This self-report inventory consists of 21 multiple-choice questions and is a widely used method to classify depression severity (55). It assesses for the presence of several symptoms related to depression, such as irritability, hopelessness and decreased cognitive performance. Physical symptoms such as weight loss and fatigue are also included. The total time required to complete this inventory is 5 to 10 min.

*Quality of Life Assessment (Short version of SF-36):* The short version of the SF-36 health survey is used as a measurement of quality of life. It provides a profile of functional health and well-being scores. It is also used as a psychometrical index of physical and mental health (56).

*Satisfaction with Life (SWL)*—is a short and rapid 7-point Likert scale that measures life satisfaction in the perspective of subjective well-being. Scores in SWL have been positively correlated with measures of mental health and also predictive of future maladaptive behaviors such as suicide attempts (57, 58).

*The Pittsburgh Sleep Quality Index (PSQI)*: a 19-item, self-report measure to evaluate overall sleep quality, primarily designed to evaluate sleep disturbance in patients with psychiatric disorders. The PSQI evaluates sleep quality in 7 categories: subjective sleep quality, sleep latency, sleep duration, habitual sleep efficiency, sleep disturbances, use of sleep medication, and daytime sleep dysfunction (59, 60).

*State-Trait Anxiety Inventory (STAI-Y) –* a self-report measure is organized in two subscales: State Anxiety Scale (S-Anxiety) wish measures the current (“right now”) presence and severity (state) of anxiety; and the Trait Anxiety Scale (T-Anxiety) which measures the general propensity to be anxious (trait). This measures focus on several domains that characterizes anxiety, such as subjective feelings, and levels of arousal (activations of the autonomic nervous systems). The trait subscale evaluates the more stable aspects of anxiety (“anxiety proneness”), such for instance worry, confidence and security (61).

*Beck Anxiety Inventory (BAI):* is a 21-item measure of anxiety focusing on somatic symptoms of anxiety. Each item is descriptive of somatic, subjective or panic-related symptoms of anxiety. Administration of BAI takes between 5 to 10 min. Since BAI focus on a “pure” measure of anxiety such as nervousness, dizziness, inability to relax, etc.), it helps discriminating between depression and anxiety (62, 63).

#### Data Management and Access to Data

Data forms and questionnaires will be coded in a standardized manner, and double-entered in a protected excel sheet. Personal information and all data collect will be kept in locked cabinets that only the principal investigator will have access. A key to access these cabinets will be kept in a safe place with limited access. Only researchers involved in the study and any public health and safety authorities will have access to the data collected in the study. Any information linking data back to the participant will be discarded to ensure that the data are truly anonymous. Data destruction will be conducted 5 years after the study has ended. Data sharing will only be possible after an agreement. The data will be stored and managed following the GDPR in the EU. According to national regulations and the Ethics Committee approval, no Data Monitoring Committee (DMC) will accompany this study.

### Statistical Analysis

#### Primary Outcome

A mixed model ANOVAs will be used to assess the clinical effects for primary outcome measure (MADRS), with intervention as between subject factor (active vs. sham tDCS), and time as within subject factor (weeks 2, 4, 8, 12, and 6 months). If there are significant baseline differences across the groups, those covariates will be included in the analysis (such as severity, anxiety, and quality of life). If significant main effects and/or interactions arise from the main analysis, *post-hoc* analysis corrected for multiple comparisons will be performed. If our hypothesis of prefrontal inter-hemispheric imbalance (as assessed by alpha power EEG) is confirmed, we will then conduct correlation analysis in order to assess whether these brain signatures correlate to long-term clinical effects in MDD.

#### Secondary Outcomes

##### EEG Analysis

To evaluate the degree of coupling between electrode pairs, we will use Magnitude Square Coherence as a pairwise connectivity measurement. For power spectrum, band power, and intraband mean and median analysis of the EEG frequency ranges, we will use the Fast Fourier transformation analysis, which will allow us to determine and measure the amplitude of the predominant EEG frequency, and properties in the time and frequency domains. For these two EEG analysis, we will define the following frequency bands: delta (1–4 Hz), theta (4–8 Hz), alpha (8–13 Hz), and beta (13–30 Hz) and four frequency sub-bands: low-alpha (9-10 Hz), high-alpha (10-12 Hz), low-beta (13-20 Hz), and high-beta (20-30 Hz), which can be obtained by decomposing the raw signal being generated in different areas of the brain.

Independent Component Analysis (ICA) will be used for artifact rejection. EEG changes during task will be assessed via task-related power (TRP)–i.e., TRP at a given electrode will be obtained by subtracting (log-transformed) power during a pre-stimulus reference interval from (log-transformed) power during the task. Power estimates will be obtained by squaring filtered EEG signals and then band power values will be averaged for both the pre-stimulus reference period and the task intervals. Degree of coupling between electrode pairs, will be assessed by using Magnitude Square Coherence as a pair-wise connectivity measurement.

### Ethics and Dissemination

The trial is registered with the U.S. National Library of Medicine Clinical Trials Registry: NCT03548545 (ClinicalTrials.gov) and is approved by the local ethics committee—*Subcomissão de Ética para as Ciências da Vida e da Saúde (SECVS)* – SECVS 174/2017.

### Confidentiality

All participants will be given subject identification codes composed by letters and numbers to which all the data will be linked. The file records that connect each participant to their identification number will be securely kept on University servers during the entire period of the study, and up to 5 years the study has ended.

### Dissemination Policy

Results of this study will be published in peer reviewed journals, and will be disseminated in national and international conferences and in social media. In any of the dissemination procedures, subjects will not be identified or notified about the event.

## Discussion

The current study describes a protocol for a parallel randomized, triple-blind, sham controlled clinical trial to test the synergetic clinical and electrophysiology effects of combining cognitive-behavioral therapy with transcranial direct current stimulation in drug-naïve, first-episode MDD patients.

By combining two therapies that have shown promising results in patients with MDD, we expect that the group that received CBT combined with active tDCS will have a greater reduction on MADRS scores, and will require lesser number of sessions in order for the clinical outcome to be reached. This result will have a significant impact since major depression is the second most prevalent mental disorder, which is thought to affect 163 million people worldwide (64). Furthermore, in Portugal, 7% of the population is diagnosed with depression every year (65), and suicide is responsible for more than a thousand deaths annually (66).

Despite the fact that tDCS has some promising effects on mood, it seems that it is in the combination of tDCS with other intervention that the effects are larger. For instance, in the study of Loo et al. (67), which randomized 64 patients to 15 sessions of 2 mA tDCS over 3 weeks and the study of Brunoni et al. (21), which enrolled 120 antidepressant-free patients with moderate and severe depression, tDCS has been shown to be effective in MDD. Moreover, in the study of Brunoni et al. (21), not only active tDCS was superior to sham tDCS but also the combined tDCS/sertraline was significantly more effective than in the other treatment groups in reducing depressive symptoms. Thus, the effects of tDCS seem to be enhanced by the combination with other interventions. This can be particularly important, if patient level data is taken into consideration. In a recent individual patient data meta-analysis (23) tDCS was shown to be less effective in high-resistant patients. This also reinforces the need of improving tDCS techniques so they can be effective in a broader depressed population.

We also propose to study, as secondary aim, whether CBT combined with tDCS can engage the proposed mechanistic target, of restoring the prefrontal imbalance and connectivity, by changes over resting-state and task-eliciting EEG. This trial will help to evaluate the efficacy of this combined treatment as compared to CBT alone and to evaluate bilateral alpha activity over the prefrontal cortex. Thus, we also expect to demonstrate that these interventions are able to reduce the inter-hemispheric asymmetry of alpha EEG activity toward the left hemisphere reported in patients with depression. We expect that the combined intervention will induce greater asymmetry reduction (at least 50% minimum reduction). We also expect that this inter-hemispheric imbalance reduction will be correlated with mood improvement.

Furthermore, this mechanistic approach is one of the main advantages of the current proposal. For instance, the use of EEG as an adjuvant tool to exclude neurological conditions or to help in the diagnosis of psychiatric disorders is a common practice in the clinical setting. Evidence of abnormal findings are obtained in about 64 to 68% of the EEGs performed in psychiatric patients (68). These results suggest that EEG can be a potential technique to be used as a coadjutant for the diagnosis and prognosis of several psychiatric conditions, with potential reliability to guide neural-based interventions. EEG has been shown to differentiate patients with MDD and non-depressed healthy controls. Numerous studies have shown that depressed individuals present an asymmetry of EEG alpha activity toward the left hemisphere, even among previously depressed individuals when compared to those who have never experienced clinically significant depression (7–9). Some studies have nevertheless failed to show the link between decreased left frontal activation and depression (19). It has been suggested that inconsistencies in this literature may be the result of clinical and/or methodological differences between laboratories, such as the inclusion or exclusion of co-morbidities (such as anxiety disorders), gender related differences, or the choice of EEG reference (9, 69, 70). Nonetheless, there is robust evidence suggesting that EEG alpha asymmetry is present among individuals with present or history of clinical depression, or even susceptibility to develop depression in the future (71). Additionally, alpha power asymmetry-based neurofeedback (NFB), which aims to train patients to increase right-to-left ratio by rebalancing the left hemisphere hypoactivation, has shown promising results in MDD (72, 73).

The advantages of EEG in MDD go far beyond its potential to detect differences between depressed vs. non-depressed individuals. Namely, EEG can be used to detect changes in the EEG patterns after interventions, and as such can be used to determine the efficacy of the intervention or if used also in the baseline, as an outcome predictor (74, 75). Furthermore, EEG is a direct measure of neural activity, it allows for chronometric sensitivity, has the potential to assess local and network effects, it is easy to use, cheap, and non-invasive. By using EEG, it is possible to quantify the electrical activity over specify regions of interest (power and coherence analysis) and, therefore, correlate with symptoms severity and response prognostic to specific treatments. Thus, creating brain based interventions.

These changes in EEG patterns have been used to direct several interventions, such as tDCS or TMS, however most study lack in the assessment of the real changes in EEG activity. For instance, based on this neurobiological basis, the main target for treating depressive symptoms using non-invasive brain stimulation (NIBS) techniques, such as tDCS and TMS, have been both the left (hypoactive) and right (hyperactive) DLPFC, by placing anodal (excitatory) over the left and cathodal (inhibitory) over the right DLPFC (76, 77). Using this mechanistic approach of facilitating the activation of the left DLPFC relative to the right, beneficial emotional, and cognitive effects in MDD were shown emotional and cognitive effects in MDD (18, 78, 79). Moreover, we chose to include adults with first episode of MDD only as to increase homogeneity of our study sample and thus increase internal validity of our findings. There are several advantages of studying the combination of these treatments in first episode MD drug-naive patients, as their brains were not changed by the pharmacological treatments, by the time course of the condition, or by the recurrence of MDD episodes (and other comorbidities). Second, combining tDCS and CBT are two therapies that have been shown to improve MDD. Moreover, tDCS is a safe, of easy administration, and not expensive non-invasive brain stimulation technique that has been shown to be effective in neuromodulating our target mechanism (imbalance over the DLPFC). Also, CBT is the golden standard treatment for MDD and has also shown to be able to neuromodulate brain structures involved in MDD. Thus, if we show that tDCS combined with CBT produce greater significant clinical improvements in MDD, this may reduce the global burden of MDD (for instance, by reducing the number of therapy sessions and the number of relapses and by producing larger long-term effects).

Therefore, the results of this project will further provide important insights into the mechanisms underlying MDD. In sum, we will be able to study the mechanistic reason underlying differences between the add-on treatment group vs. CBT combined with sham tDCS. We chose a population that is not very well studied, namely patients drug-naïve, first-episode MDD with mild to moderate symptoms, because several studies failed to show the link between decreased left frontal activation and depression. By using resting state EEG, it will be possible to simultaneously understand the neural effects of the intervention, as well as potential response predictors for future trials.

## Trials Status

This clinical trial is currently on the recruitment phase.

## Author Contributions

SC and JL designed and developed the study protocol. ÓFG, AB, AF-G, and FF provided inputs to the design and development of the protocol and contributed equally to this work. All authors read and approved the final manuscript. All authors contributed to the article and approved the submitted version.

## Conflict of Interest

The authors declare that the research was conducted in the absence of any commercial or financial relationships that could be construed as a potential conflict of interest.
